# The Grand SANS FLUORO (SAy No Series to FLUOROsopy) Study: Examining Fluoroscopy Use in More than 1,000 Ablation Procedures

**DOI:** 10.19102/icrm.2020.1100903

**Published:** 2020-09-15

**Authors:** Robert L. Percell, Jonathan L. Pike, Rhonda K. Olmsted, Jenna E. Beideck, Haley L. Nunes, Kellie N. Johnson, Matthew Schaffer, Lindy B. Vachok, Stacy M. Sveen, Emily J. Keim, Shirley O. Mohr-Burt, Rose M. Saalfeld, Casey A. Beran, Timothy W. Allison, John F. Stock

**Affiliations:** ^1^SANS FLUORO Institute, Electrophysiology Department, Bryan Heart Institute, Lincoln, NE, USA; ^2^Bryan Heart Institute, Lincoln, NE, USA; ^3^Abbott Medical, Abbott Laboratories, Abbott Park, IL, USA; ^4^Lake Region Healthcare, Morris, MN, USA

**Keywords:** Ablation, arrhythmia, atrial fibrillation, fluoroscopy, three-dimensional mapping

## Abstract

The majority of electrophysiologists routinely use fluoroscopy (FLUORO) during ablation procedures for common arrhythmias despite the known complications of radiation exposure and protective lead use. This study assessed the safety of catheter ablation (CA) with FLUORO versus without FLUORO (SANS FLUORO) in patients with the following common arrhythmias: atrial fibrillation (AF), atrial flutter, supraventricular tachycardia, and ventricular tachycardia. A total of 1,258 CA procedures were performed in 816 consecutive patients over a 53-month period (SANS FLUORO CA: 609 patients; FLUORO CA: 209 patients). The secondary outcome was the efficacy of AF ablation in FLUORO versus SANS FLUORO patients. Ultimately, there was no statistically significant difference found concerning the safety of CA in the SANS FLUORO and FLUORO groups in terms of procedure time, vascular complications, tamponade, stroke, or death. FLUORO patients had markedly increased FLUORO time, increased radiation exposure, and increased dose-area product (all p < 0.0001). AF development after SANS FLUORO CA of AF was not different from that after FLUORO CA regardless of the pulmonary vein isolation (PVI) modality used (cryoablation versus radiofrequency) at 24 months (p = 0.21). Additionally, women fared just as well as men after CA ablation for AF. At 36 months, 58% of SANS FLUORO AF device patients were free from AF. As such, SANS FLUORO CA of common arrhythmias appears to be as safe as FLUORO CA but with a markedly reduced level of radiation exposure. Also, SANS FLUORO CA remains as effective as FLUORO CA in the prevention of AF for up to 24 months.

## Introduction

Catheter ablation (CA) is the recommended treatment for most symptomatic arrhythmias, especially if medical treatment fails.^1^ Electrophysiological procedures have experienced the largest increase in number in the field of cardiology in the past five years and are only expected to become more frequent.^2^ The majority of electrophysiologists (EPs) who perform interventional procedures for patients who have symptomatic arrhythmias frequently rely on fluoroscopy (FLUORO) despite having multiple visualization modalities at their disposal, including three-dimensional mapping techniques, intracardiac ultrasound (ICE), and remote magnetic navigation systems.^3–9^ These procedures require patients to be exposed to potentially harmful fluoroscopic radiation at a level that is not trivial.^10–13^ While as low as reasonably achievable (ALARA) is the most common concept that all EPs attempt to adhere to,^13–15^ no amount of fluoroscopic radiation has been deemed to be completely safe. Frequent complications of radiation exposure are well-known and include both deterministic and stochastic (dose-related and dose-independent, respectively) effects such as local erythema, cataracts, skin desquamation, leukopenia, organ atrophy, birth defects, and various organ and bone cancers.^16,17^ Furthermore, wearing lead protection to guard against radiation leads to a plethora of orthopedic problems.^10,11,16^ Among all cardiologists, EPs have slightly more back, neck, or leg problems than their interventional, pediatric, or general cardiology peers, which has been labeled as the “scourge” of the profession.^14^

Only recently, a “revolution” has occurred in that there are an increasing number of studies demonstrating the safety of zero-radiation or no-FLUORO (SANS FLUORO).^5,18–28^ However, to date, the majority of SANS FLUORO studies have been performed in the pediatric population and most studies in adults include only a limited number of nonrandomized patients or case reports.^29–33^ Further, the majority of these studies have short-term follow-up periods less than 12 months. The intermediate- and long-term efficacy of fluoroless ablations [especially in atrial fibrillation (AF)] is largely yet to be determined.

We describe our experience with a number of retrospective cases that were performed initially with FLUORO followed by SANS FLUORO as our laboratory transitioned to a near-fluoroless laboratory. This series includes the following ablations for common arrhythmias: radiofrequency (RF) and cryoablation for AF and atrial flutter (AFL); supraventricular tachycardias (SVTs) including AV node reentry tachycardia (AVNRT), AV reentry tachycardia (AVRT), and atrial tachycardia (AT); AV nodal ablations; premature ventricular contractions (PVCs); and ventricular tachycardia (VT).

To our knowledge, this series is the largest presentation to date of observational, retrospective data from more than a single site and which boasts the longest follow-up period in a nonacademic, community setting. Further, this investigation included multiple clinical variables and the largest number of device patients as well as specific ablation data derived from current contact-force sensing and cryoballoon catheters. Data in this study were analyzed with respect to both safety and efficacy.

The primary objective of this study was to assess the safety of CA with FLUORO versus SANS FLUORO in patients with the following common arrhythmias: AF, AFL, SVT, and VT. The secondary objective was to determine the efficacy of AF ablation in FLUORO and SANS FLUORO patients, respectively.

## Methods

Retrospective data from two separate, community, nonacademic settings comparing a total of 1,258 procedures in 816 consecutive patients were included. A total of 227 patients underwent procedures that were performed with FLUORO and 609 patients underwent SANS FLUORO procedures, respectively. Patients were indexed according to their primary clinical arrhythmia. Clinical variables were obtained directly from the electronic health records. Safety and efficacy were examined. All statistical analyses were performed using the Statistical Package for the Social Sciences version 21.0 software program (IBM Corp., Armonk, NY, USA). Student’s t-tests were used to compare numerical values, while chi-squared tests were used to compare categorical variables. A p-value of less than 0.05 was considered to be statistically significant (two-sided).

**Figure 1:**
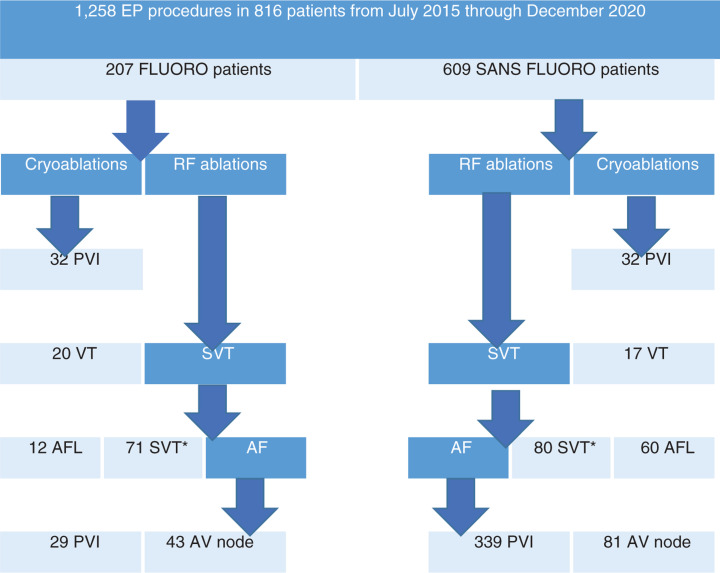
Patient flow in the Grand SANS FLUORO study. Patients were classified based on their presenting primary clinical arrhythmia and were counted only once per laboratory visit. SVT patients included those with AVNRT, AVRT, and AT. VT patients included PVC patients. AF: atrial fibrillation; AFL: atrial flutter; AV: atrioventricular; EP: electrophysiology; FLUORO: fluoroscopy; PVI: pulmonary vein isolation; SANS FLUORO: without fluoroscopy; SVT: supraventricular tachycardia; VT: ventricular tachycardia.

Patients were divided into two groups: SANS FLUORO and FLUORO. Patients were studied from July 9, 2015 to December 31, 2019. Kaplan–Meier curves were generated for AF-free survival among AF patients. AF was defined as continuous AF of more than five minutes or greater than 1% of the total time on device interrogations. The average follow-up period was 24 months **(Figure 1)**. Informed consent was obtained prior to each procedure and all patients were informed about the risks, benefits, and alternatives of SANS FLUORO CA. Regular scheduled electrocardiographic follow-up for efficacy was conducted at two weeks, three months, six months, one year, 18 months, and 24 months via Holter monitoring if patients did not have an implantable device [eg, loop recorder, pacemaker, or implantable cardioverter-defibrillator (ICD)]. For device patients, a total follow-up duration of 36 months was documented. Safety parameters collected included hospital duration, vascular complications requiring surgical intervention, tamponade, intraoperative death, and stroke while hospitalized. Patients were excluded from this study if they did not undergo ablation procedures.

**Figure 2:**
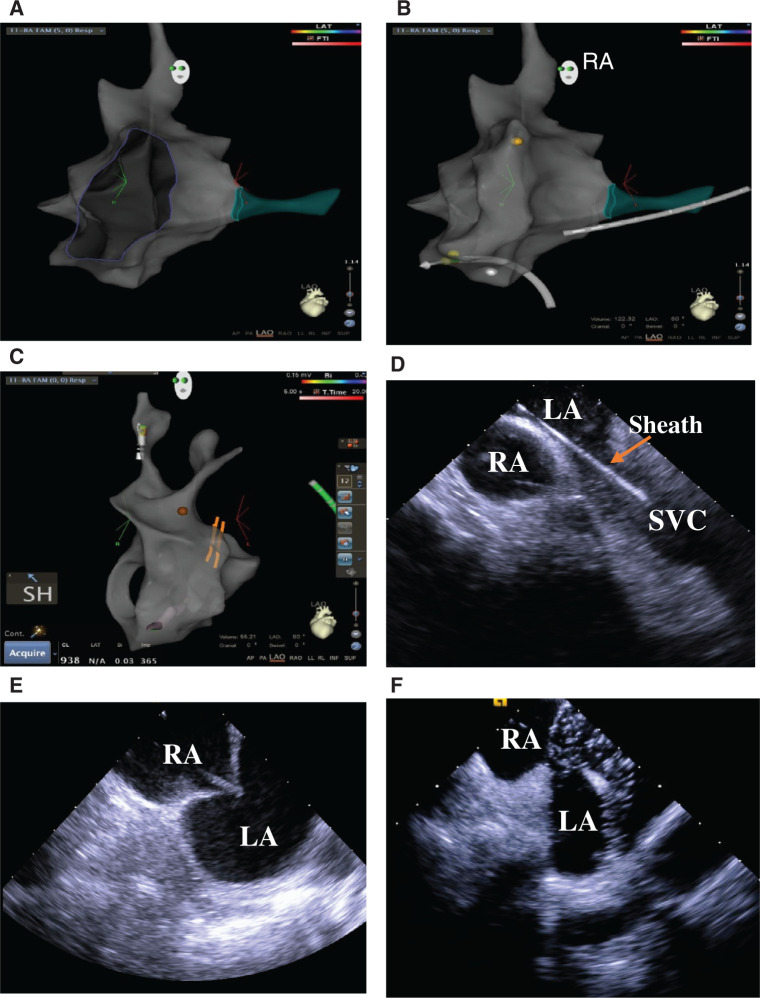
SANS FLUORO technique. **A:** RA fast anatomical map with the coronary sinus shown in green and the tricuspid valve cut away. **B:** RA fast anatomical map with coronary sinus catheter and His bundle shown in yellow as well as the ablation catheter. **C:** Sheath placement in the SVC is noted by two dark bands and the SH. **D:** Sheath in the SVC on ICE. **E:** Tenting of the intra-atrial septum on ICE. **F:** Placement of the sheath into the LA with confirmation of bubbles in the LA. LA: left atrium; RA: right atrium; SH: sheath.

Patients underwent EPS or ablation with either monitored anesthesia care or general anesthesia. All SANS FLUORO procedures used either the EnSite Precision™ cardiac mapping system (Abbott Laboratories, Chicago, IL, USA) or the CARTO^®^ 3 mapping system (Biosense Webster, Diamond Bar, CA, USA). Patients underwent ablation manually or using the Niobe^®^ robotic magnetic navigation system (Stereotaxis, St. Louis, MO, USA). A full description of the SANS FLUORO has been presented elsewhere.^8,33–35^ Briefly, after sheaths were placed, three-dimensional electroanatomic maps of the inferior vena cava, right atrium (RA), right ventricle (RV), and superior vena cava (SVC) as well as the chamber of interest if the ablation was left-sided [eg, pulmonary vein (PV) isolation (PVI), left VT, or left ventricular outflow tract PVC] were generated. Only left-sided lesions requiring transseptal crossings were performed, with ICE conducted by an additional technician **(Figure 2)**. All RF PVIs were performed with contact force-sensing catheters. Intravenous heparin was administered to maintain the activated clotting time (ACT) between 250 and 300 seconds. At the end of the procedure, sheaths and catheters were removed and either manual pressure, closing device, or figure-of-eight suture when the ACT was less than 180 seconds was adopted for hemostasis. The procedure time was measured as the total time spent in the room including for anesthesia administration.

## Results

Patients in the SANS FLUORO group were heavier (101.8 kg versus 92.5 kg; p = 0.0001), more likely to be male (60.5% versus 52.7%; p = 0.033), and more likely to have paroxysmal arrhythmias (37.1% versus 29.5%; p = 0.047) than FLUORO patients. Additionally, SANS FLUORO patients were less likely to have coronary artery disease (21.7% versus 33%; p = 0.0008), had higher ejection fractions (51% versus 47%; p = 0.0008), and were more likely to require cardioversion after the procedure (21% versus 11%; p = 0.0015) than FLUORO patients. Finally, SANS FLUORO patients were more likely to prescribed a novel anticoagulant (NOAC), less likely to be prescribed a vitamin K antagonist, and less likely to have an implanted device (ie, pacemaker or ICD) than FLUORO patients (all p < 0.0001) **(Table 1)**.

SANS FLUORO patients received markedly less FLUORO, radiation exposure, and dose-area product than FLUORO patients (all p < 0.0001). SANS FLUORO patients had overall increased RF ablation times (p < 0.0001) than FLUORO patients, but the overall procedure times (p = not significant) and hospital stay lengths (p = 0.085) of both groups were not significantly different. The vascular complication rates in both groups were low at 0.5% in the FLUORO group and 0.3% in the SANS FLUORO group, respectively, without a significant difference (p = 0.75). Tamponade rates did not significantly vary at 1% in the FLUORO group and 1.2% in the SANS FLUORO group, respectively (p = 0.83). No patients experienced stroke or died in the FLUORO or SANS FLUORO group **(Table 2)**.

Across all arrhythmia types, SANS FLUORO patients experienced significantly reduced FLUORO times, dose-area products, and exposure times (all p < 0.001) than FLUORO patients. The RF ablation time was significantly shorter among RF PVI SANS FLUORO patients than among RF PVI FLUORO patients (2,828 versus 3,428 seconds; p < 0.0001). Additionally, SVT SANS FLUORO patients spent a significantly shorter amount of time in the hospital (0.76 versus 1.2 days; p = 0.0251) relative to SVT FLUORO patients. Patient undergoing RF PVI or VT ablations experienced major complications but the occurrence of such was not significantly different overall between the SANS FLUORO and FLUORO patients based on arrhythmia type (p = 0.37) **(Table 3)**.

**Figure 3:**
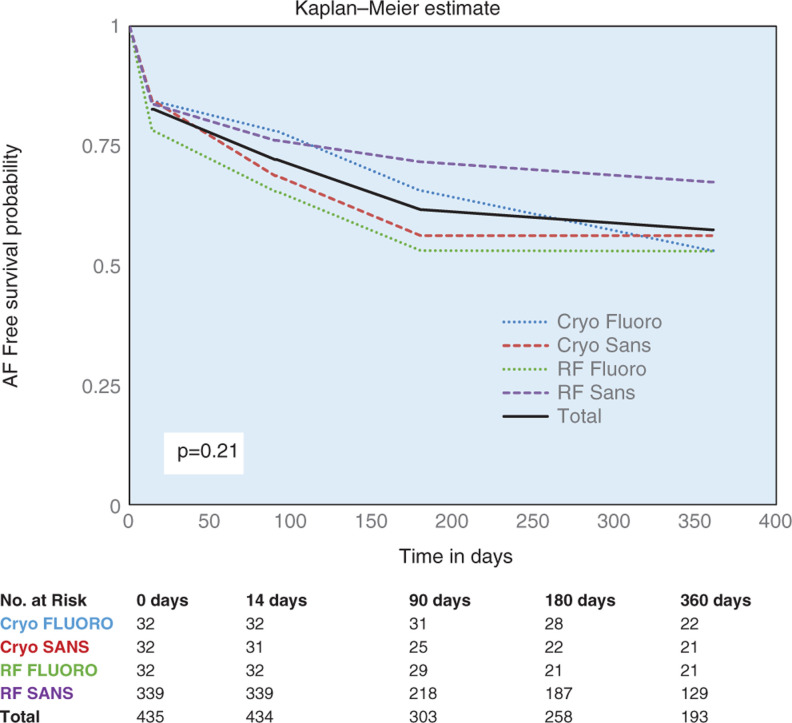
PVI efficacy. Kaplan–Meier estimates of AF-free survival within 360 days of PVI ablation procedure by the following PVI modalities: cryoablation with FLUORO, cryoablation SANS FLUORO, RF ablation with FLUORO, and RF ablation SANS FLUORO. Cryo FLUORO: cryoablation with fluoroscopy; Cryo SANS: cryoablation without fluoroscopy; RF FLUORO: radiofrequency ablation with fluoroscopy; RF SANS: radiofrequency ablation without fluoroscopy.

There was no difference in the rate of development of AF following CA between the SANS FLUORO and FLUORO groups at 24 months (p = 0.21). Additionally, no difference in AF development was seen between the cryoablation and RF SANS FLUORO and FLUORO groups. There was a trend toward less frequent AF development among SANS FLUORO RF PVI patients than among FLUORO RF PVI patients after CA (p = 0.09) **(Figure 3)**.

A meticulous scrutinization of the RF ablation data revealed that SANS FLUORO AF patients underwent fewer total ablations (162 versus 167 ablations; p = 0.0236) and were subjected to greater overall contact force (15.1 versus 10.98 g) and, therefore, higher force–time integral (FTI) values (221.52 versus 152.74 g/s; p < 0.0001) than FLUORO patients **(Table 4)**.

**Figure 4:**
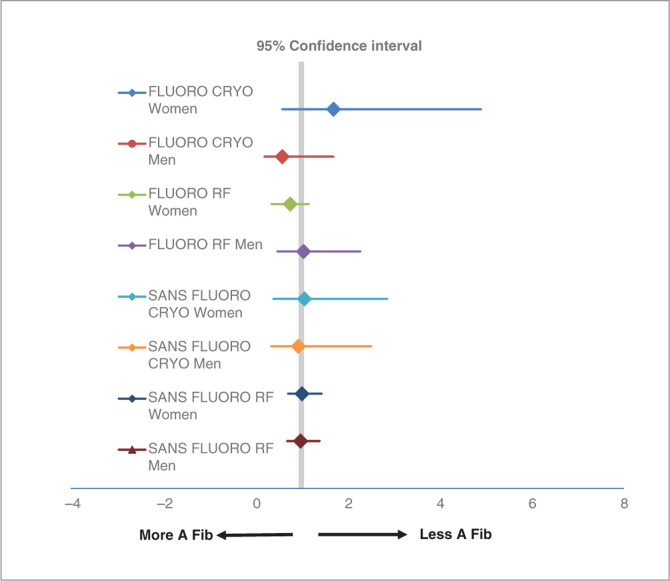
The odds ratios of AF development based on sex as well as AF ablation modality. CRYO: cryoablation. FLUORO: fluoroscopy; CRYO: cryoablation; RF: radiofrequency; SANS FLUORO; without fluoroscopy.

There were no differences in the occurrence of AF development based on sex. Regardless of PVI modality and FLUORO or SANS FLUORO, both men and women fared similarly with cryoablation and RF ablation **(Figure 4)**.

**Figure 5:**
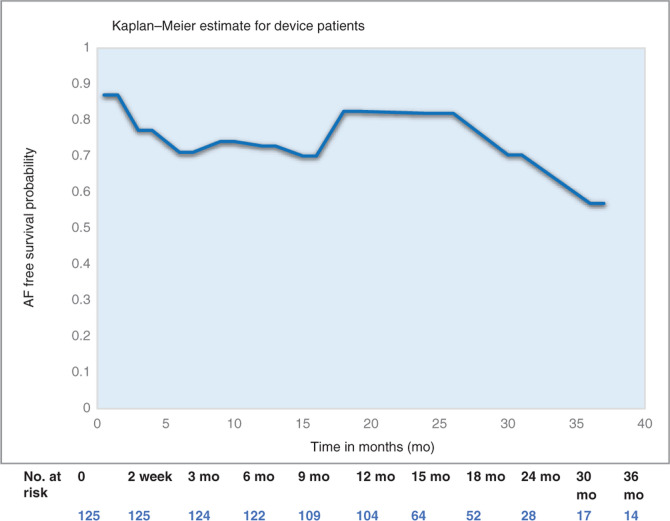
PVI efficacy in device patients. Kaplan–Meier estimates of AF-free survival in device patients within 36 months of PVI ablation. All patients had dual-chamber devices (pacemakers or ICDs) or implantable loop recorders. AF was defined as greater than 1% AF burden or an episode lasting more than five minutes.

In patients with implantable devices (dual-chamber pacemakers and ICDs as well as implantable recorders), more frequent and longer-term follow-up data were obtainable. At 36 months, 58% of SANS FLUORO AF patients were free from AF **(Figure 5)**.

## Discussion

The evaluation of a therapeutic value of a particular treatment strategy should include the consideration of the risk–benefit relationship. First and foremost, the main results of this series demonstrate that SANS FLUORO CA is as safe as FLUORO CA in the treatment of most common arrhythmias (ie, AF, AFL, SVT, and VT) without additional risk. Second, AF SANS FLUORO CA patients had similar intermediate-term efficacy relative to FLUORO CA patients, which was especially observed in device patients out to 36 months. Finally, women fared just as well as men after CA ablation for AF, regardless of PVI modality (RF or cryoablation) or FLUORO or SANS FLUORO.

Patients in the SANS FLUORO group showed increased RF ablation times overall when compared with FLUORO patients, which may be explained by the fact that that there were significantly more AV node ablations in the FLUORO group than in the SANS FLUORO group (21% versus 13%; p = 0.01). AV-node CA is less complex and requires much less RF energy than RF CA for PVI. The SANS FLUORO group had approximately twice the number of PVI patients relative to the FLUORO group (60% versus 29.5%; p < 0.0001). As more patients in the FLUORO group underwent AV-node ablation, they also had more devices (21% versus 13%) than those in the SANS FLUORO group. Patients were not “preselected” because they had devices in place prior to the procedure.

Major complications were seen in left-sided ablations only. Similar to in previous large studies, tamponade rates were 1% and 3% in the SANS FLUORO and FLUORO groups, respectively, with no statistically significant difference. Notably, more patients in the FLUORO group than in the SANS FLUORO group had tamponade. The tamponade rate in the VT FLUORO subgroup at about 6% appears unusually elevated; however, the size of this subgroup was quite small at 17 patients. There were no deaths or strokes in any patients during the hospitalization period. It should also be highlighted that 84% of SANS FLUORO patients versus 52% of FLUORO patients were prescribed NOACs, with no increase in bleeding events. Furthermore, no patients in either group experienced device lead dislodgement; of particular interest, one patient in the SANS FLUORO group with a total of six intracardiac leads underwent a successful, uncomplicated PVI procedure.

In terms of safety, all patients in the SANS FLUORO group experienced strikingly less radiation exposure when compared with those in the FLUORO group. Even in the rare tamponade patient, FLUORO time, radiation exposure, and dose-area product were resoundingly reduced in the SANS FLUORO group. One patient in the SANS FLUORO group was morbidly obese at 226.8 kg with a body mass index of 70 kg/m^2^ and could not be placed on the FLUORO table, which required the AFL ablation to be performed in a bariatric bed.^36^ In total, FLUORO patients received 272 mGy, which may be the typical therapeutic radiation dose for some body parts. These findings were achieved while practicing ALARA and included remote navigation procedures in which patients may have only received a total of 30 seconds of FLUORO in the FLUORO group. Notably, previous studies have demonstrated increased FLUORO times and levels of radiation exposure in AF cryoballoon PVI patients when compared with in RF PVI patients^37^; our findings in the SANS FLUORO patients imply that both techniques can be performed equally without exposure.

During ablation, SANS FLUORO RF PVI patients had higher average contact force and FTI values, but the distinct clinical benefit was unclear as there was no difference in overall AF-free efficacy at one year as compared with among FLUORO RF PVI patients. Additionally, in AF patients, regardless of modality type (RF or cryoablation) and SANS FLUORO or FLUORO, the rates of AF development postablation were similar at 24 months. SANS FLUORO device patients showed sustained AF-free rates of 80% at 24 months and 58% at 36 months. These findings are consistent with the findings of previous persistent AF studies examining efficacy post-CA.^37,38^

Both women and men fared equally well after CA for AF regardless of PVI modality. This finding is in contrast with those of other studies that demonstrated that female sex may be the largest predictor of AF recurrence following PVI.^39^ This outcome should be considered with the knowledge that, usually, most studies still do not include assessments of comparative sex-specific efficacy, safety, and procedural characteristics due to the unequal and low enrollment numbers of women.^40^ This series had a relatively higher percentage of women, with 47% in the FLUORO group and 39% in the SANS FLUORO group; the comparatively increased female-to-male ratio may have contributed to the similar efficacy findings.

### Safety consideration

Despite multiple small series and a paucity of large randomized trials, SANS FLUORO ablation techniques have not been widely adopted. Right-sided arrhythmias may be easily approached with little additional training. Transseptal crossing for left-sided arrhythmias require high-quality ICE imaging. ICE is crucial to eliminating radiation exposure and may require the involvement of an additional technician or physician (ie, fellow). All PVs were identified using only ICE in every patient. Only two patients underwent preprocedure computed tomography (CT) imaging (both receiving PVIs for the third time). A single patient in whom the right inferior PV could not be seen underwent a postprocedure CT scan that revealed that the vein was previously ligated during a partial pneumonectomy for lung cancer. The long-term safety effects for patients and staff from the elimination of radiation exposure and the removal of protective lead aprons are clearly apparent.

### Limitations

The most apparent limitation of this study is that it is not randomized. At this point, we believe that performing FLUORO procedures would place patients at an unnecessarily increased radiation risk without proven clinical benefit, similar to sham trials of CA of AF. Additionally, the number of patients in the FLUORO group was approximately one-third that in the SANS FLUORO group. This may have introduced unintended bias. Furthermore, most patients were men. Meanwhile, though various ethnic groups have been reported to have different rates of AF development,^36,41^ this study is still expected to be applicable as excess radiation exposure is detrimental to all. Additionally, this study examined multiple arrhythmia types with different pathophysiologic mechanisms and only CA techniques using RF and cryoablation modalities were included. Finally, longer-term follow-up would offer additional insight. Finally, a cost analysis was not specifically performed; however, previous studies have indicated that using mapping systems in the CA of AF is cost-effective, although this may not be the case for SVT.^42^

### Clinical implications

With the ever-increasing prevalence of common arrhythmias (AF, AFL, SVT, and VT) combined with the limited efficacy of rate-controlling medications and antiarrhythmic drugs, CA has become the preferred treatment and, in the case of some arrhythmias, the cure.

Strategies to improve the safety of CA for arrhythmias continue to evolve, such as reductions in FLUORO or the use of SANS FLUORO techniques, which eliminate the need for ionizing radiation. In our series, there appears to be no increased risk of major complications such as vascular injury, tamponade, stroke, and death; further, there is also no loss of demonstrable efficacy as noted in AF patients. Additionally, women and men showed similar outcomes in terms of AF recurrence throughout this study. Future larger, possibly randomized studies comparing SANS FLUORO and FLUORO ablation may provide additional information about clinical efficacy and long-term follow-up to define the durability of treatment effect and further elucidate the efficacy and safety profiles.

Reducing or eliminating FLUORO could help to eliminate harmful radiation-induced complications in patients and decrease issues among EPs and laboratory staff without compromising overall efficacy.

## Conclusions

In this patient population, SANS FLUORO CA ablations for common arrhythmias were safe. There appears to be no added risk with the obvious benefit of the elimination of harmful ionizing radiation. Additionally, SANS FLUORO CA is as effective as FLUORO CA in preventing AF at 24 months. Finally, women who underwent CA for AF had similar AF-free rates as men, regardless of treatment modality.

## Figures and Tables

**Table 1:** Clinical Characteristics of Patients in the Grand SANS FLUORO Study

Variable	FLUORO	SANS FLUORO	p-value
Age, years	67.16	64.53	0.104
Weight, kg	92.53	101.8	0.0001*
Male sex	0.527	0.605	0.0332*
White race	0.971	0.994	0.8732
Hypertension	0.657	0.632	0.5208
Diabetes mellitus	0.213	0.182	0.3371
Paroxysmal AF	0.295	0.371	0.0467*
Persistent AF	0.551	0.502	0.23
CAD	0.333	0.217	0.0008*
CABG	0.09	0.059	0.1053
AAD	0.478	0.634	0.0001
β-blocker	0.836	0.813	0.4593
Amiodarone	0.275	0.335	0.1122
ACE/ARB/Entresto	0.343	0.368	0.5208
H/o CV	0.50	0.491	0.776
EF	0.477	0.51	0.0008*
CV post	0.111	0.21	0.0015*
LA size, mm	41.575	41.864	0.6906
NOAC	0.527	0.837	< 0.0001*
Warfarin	0.193	0.092	< 0.0001*
Pacer	0.256	0.082	< 0.0001*
ICD	0.179	0.052	< 0.0001*

AAD: antiarrhythmic drugs; ACE/ARB: angiotensin-converting enzyme/angiotensin receptor blocker; CABG: coronary artery bypass graft; CAD: coronary artery disease; CV: cardioversion; EF: ejection fraction; FLUORO: with fluoroscopy; H/o: history of; ICD: implantable cardioverter-defibrillator; LA: left atrium; NOAC: novel oral anticoagulant; SANS FLUORO: without fluoroscopy.

*Statistically significant.

**Table 2:** Safety Results of Patients in the Grand SANS FLUORO Study

	FLUORO	SANS FLUORO	p-value
**FLUORO** time, min	9.9228	0.0038	< 0.0001*
DAP, μGym^2^	1508.3545	0.02622	< 0.0001*
Exposure, mGy	271.9598	0.2874	< 0.0001*
Ablation time, s	1066.4458	1879.9084	< 0.0001*
Procedure time, min	196.5922	197.6403	0.9308
Hospital stay, days	1.4559	1.2374	0.0847
Vascular complications	0.0048	0.00328	0.7507
Tamponade	0.0096	0.0115	0.8274
CVA	0	0	NA
Death	0	0	NA

AAD: antiarrhythmic drug; CVA: cerebrovascular accident; DAP: dose area product; FLUORO: with fluoroscopy; mGy: milligray; SANS FLUORO: without fluoroscopy; μGym^2^: microgray × meters^2^.

*Statistically significant.

**Table 3:** Safety Results of the Grand SANS FLUORO Patients Based on Ablation Type

	SVT		RF PVI		Cryo PVI		VT		p-Value
	FLUORO	SANS FLUORO	FLUORO	SANS FLUORO	FLUORO	SANS FLUORO	FLUORO	SANS	
FLUORO time, min	8.883	0	8.296	0.0002	21.797	0	2.015	0.012	< 0.001*
DAP, μGym^2^	328	0	3,920.772	0.0002	1,447.125	0	169.409	0	< 0.0001*
Exposure, mGy	126.3636	0	362.8642	0.0002	483.803	0	71.1	0	< 0.0001*
Ablation time, s	275.652	276.72	3,428.036*	2,828.12*			1,653.44	1,320.07	< 0.0001*
Procedure time, min	168.75	152.02	228.43	223.71	192.72	210.3	385.2	239.59	NS
Hospital stay, days	1.208*	0.756*	1.143	1.236	1.063	1.061	2.2	1.59	0.0251*
Vascular complication	0	0	0	0.006	0	0	0.059	0	0.1848
Tamponade	0	0	0.03125	0.0179	0	0	0	0.059	0.3714
CVA	0	0	0	0	0	0	0	0	NA
Death	0	0	0	0	0	0	0	0	NA

CVA: cerebrovascular accident; Cryo: cryoablation; DAP: dose area product; FLUORO: with fluoroscopy; NS: not significant; PVI: pulmonary vein isolation; SANS FLUORO: without fluoroscopy; VT: ventricular tachycardia; μGym^2^: microgray × meters^2^.

*Statistically significant.

**Table 4:** RF PVI Ablation Data of the Grand SANS FLUORO patients

	FLUORO	SANS FLUORO	p-value
No. of ablations, s	166.7083	152.0489	0.0236*
Duration per ablations, s	19.8532	15.6681	0.1506
Force, g	10.9782	15.1296	< 0.0001*
Maximum temperature, °C	41.2917	39.7356	0.0073*
Maximum power, W	38.88	38.2413	0.4523
Minimum power, W	27.2	27.061	0.9029
Base impedance, Ω	132.2617	135.837	0.2982
Impedance drop, Ω	6.5963	7.264	0.5816
FTI, g/s	152.7369	221.5157	< 0.0001*
LSI	NA	4.8229	NA
AI	NA	483.915	NA

AI: ablation index; FLUORO: with fluoroscopy; FTI: force–time integral; LSI: lesion size index; PVI: pulmonary vein isolation; NA: not applicable; RF: radiofrequency; SANS FLUORO: without fluoroscopy.
